# Identification of runner fatigue stages based on inertial sensors and deep learning

**DOI:** 10.3389/fbioe.2023.1302911

**Published:** 2023-11-17

**Authors:** Pengfei Chang, Cenyi Wang, Yiyan Chen, Guodong Wang, Aming Lu

**Affiliations:** ^1^ School of Physical Education and Sports Science, Soochow University, Suzhou, China; ^2^ Department of Physical Education, Suzhou Vocational University, Suzhou, China

**Keywords:** fatigue stages, runner, inertial sensors, deep learning, sports biomechanics

## Abstract

**Introduction:** Running is one of the most popular sports in the world, but it also increases the risk of injury. The purpose of this study was to establish a modeling approach for IMU-based subdivided action pattern evaluation and to investigate the classification performance of different deep models for predicting running fatigue.

**Methods:** Nineteen healthy male runners were recruited for this study, and the raw time series data were recorded during the pre-fatigue, mid-fatigue, and post-fatigue states during running to construct a running fatigue dataset based on multiple IMUs. In addition to the IMU time series data, each participant’s training level was monitored as an indicator of their level of physical fatigue.

**Results:** The dataset was examined using single-layer LSTM (S_LSTM), CNN, dual-layer LSTM (D_LSTM), single-layer LSTM plus attention model (LSTM + Attention), CNN, and LSTM hybrid model (LSTM + CNN) to classify running fatigue and fatigue levels.

**Discussion:** Based on this dataset, this study proposes a deep learning model with constant length interception of the raw IMU data as input. The use of deep learning models can achieve good classification results for runner fatigue recognition. Both CNN and LSTM can effectively complete the classification of fatigue IMU data, the attention mechanism can effectively improve the processing efficiency of LSTM on the raw IMU data, and the hybrid model of CNN and LSTM is superior to the independent model, which can better extract the features of raw IMU data for fatigue classification. This study will provide some reference for many future action pattern studies based on deep learning.

## 1 Introduction

Running is one of the most popular and widely practiced sports in the world, but it carries a significant risk of pain and injury (Gholami et al., 2020). As the number of people involved in running continues to increase, studies have shown that up to 70% of regular runners suffer from a running-related sports injury (Mitchell et al., 2015a). Running fatigue causes changes in normal gait parameters and increases the risk of injury. In order to reduce the number of injuries and increase the benefits of running, the running load, represented by the accumulation of fatigue, needs to be managed appropriately (Andarawis-Puri and Flatow, 2011). Accurate and real-time detection of fatigue during running activity can be employed to provide feedback to runners to avoid over training and excessive loading that can lead to neuromuscular injury.

Fatigue is a multi-factorial and complex phenomenon that affects how an individual performs an activity. It is a key symptom in defining frailty and aging problems, and in many cases, it is also a major factor in inefficiency and reduced quality of life. During the last decade, many researchers have been interested in the use of different methods to assess fatigue (Lambay et al., 2021). Under laboratory conditions, fatigue assessment is typically performed using 3D motion capture systems, which are expensive and obstructive owing to the application of skin-mounted markers. In addition, data processing can be time consuming and often requires specific expertise to interpret the processed data and make recommendations regarding the observed results. In real-world scenarios, most fatigue identification and management is based on subjective scales of fatigue perception (Winter, Gordon, and Watt, 2017), and one of the most commonly applied protocols in physical monitoring is the Borg Rating Perceived Exertion and Fatigue (RPE) Scale, where a lower number represents a non-fatigued state and a higher number represents an exhausted state (Scherr et al., 2013). This method can quantitatively characterize the degree of fatigue. However, as with most questionnaire-based methods, recall bias and validity deficiency can be unavoidable in such subjective approach (Soriano-Maldonado et al., 2014).

The field of wearable technology has made tremendous progress in recent years, with breakthroughs in cost, ease of wear, and wear interference, and is now the primary device for measuring physical activity (Hilty et al., 2021). Although researchers have conducted numerous studies on human motion analysis and action recognition, studies on fatigue prediction and estimation are still limited (Jiang et al., 2021a). In contrast to the subjective and discontinuous RPE method, inertial measurement units (IMUs) can provide dynamic data related to motor fatigue, which contains kinematic and kinetic information that can reveal changes due to physical fatigue (Harper et al., 2022). Unlike action recognition, fatigue gait is a different pattern of the same type of action and requires a more nuanced understanding of the action. Most of the existing research has been tested in laboratory scenarios, and there is a lack of applied research in real outdoor scenarios (Taylor-Covill and Eves, 2013; Fohrmann et al., 2022). Additionally, most of the studies on fatigue monitoring and classification adopt traditional classifiers for simple binary classification, while from the perspective of practical applications, not only the determination of fatigue or not, but also the generation and development of fatigue should be considered to identify the fatigue level (Delgado-Álvarez et al., 2022). Meanwhile, existing studies have mostly used feature engineering of a single IMU to construct datasets, and there is no multi-classification dataset based on raw time series for the time being (Garcia-Gonzalez et al., 2020). In addition, it is not clear to what extent running fatigue affects each part of the lower extremity and its representation on IMU data, and it is necessary to construct a multi-classification running fatigue database based on multi-site IMU.

Machine learning models, especially some recently proposed deep learning methods, provide prediction possibilities for a wider range of domains. The development of deep neural networks makes it possible for learning models to directly manipulate the raw data, thus reducing the workload of feature extraction, i.e., features and the extraction process based on empirical judgments are no longer necessary (Tunca et al., 2020). Different from traditional machine learning methods, with deep learning, we can greatly reduce the workload of feature production, and the model can automatically learn deeper and more advanced hidden features in the data by training an end-to-end neural network. In addition to traditional classifiers, optimized deep learning models can be built from IMU data features to evaluate driving fatigue action patterns (Zhang et al., 2021).

It is worth emphasizing that in the development of deep models, the deep models commonly used for action pattern recognition include Convolutional Neural Network (CNN), Long Short-Term Memory Network (LSTM), Attention Mechanism (Attention) and its hybrid models (Ye et al., 2021; Wang, 2022). CNN can extract effective features from signals and has achieved good results in speech recognition, image classification, and text analysis. It has been shown that CNN can retain the correlation between the before and after signals in human action recognition relative to other models when classifying time-series data in human action recognition (Wang et al., 2022). LSTM is a typical representative of recurrent neural networks, which has been widely used in fields such as handwriting recognition (Graves et al., 2009), character generation (Ergen and Kozat, 2017), automatic language translation (Tang et al., 2020), speech recognition (Coto-Jiménez, 2019), video processing (Song et al., 2019), and so on. Recently, LSTM has gradually gained attention in gait and action recognition, and researchers have mainly used machine vision and wearable sensors to explore its applications (Ordóñez and Roggen, 2016).

Therefore, this study constructs fatigue prediction deep models of independent single/dual layer LSTM, CNN, LSTM with attention mechanism, and hybrid model of CNN and LSTM, respectively, in an attempt to establish a modeling approach for IMU-based subdivided action pattern evaluation and explore the classification performance of different deep models for predicting running fatigue.

## 2 Materials and methods

### 2.1 Subjects and data collection

Nineteen healthy male runners (age: 28.2 ± 4.8 years, height: 174.6 ± 5.7 m, and body mass: 72.4 ± 5.3 kg) were selected for the experiment; subjects were free of significant physiological disorders and musculoskeletal injuries. Subjects were informed of the experimental protocol, signed an informed consent form before participating in the test, and consented to the release of their images or videos in publications related to this experiment. All subjects had a running habit for more than 12 months, with an average of more than 2 times per week and an average weekly running volume of more than 10 km. The best performance of 10 km within 6 months before the test was counted and the average speed was calculated. The study protocol was approved by the College Human Research Ethics Committee (20211227H03) and written informed consent was obtained from all subjects prior to participation.

**FIGURE 1:**
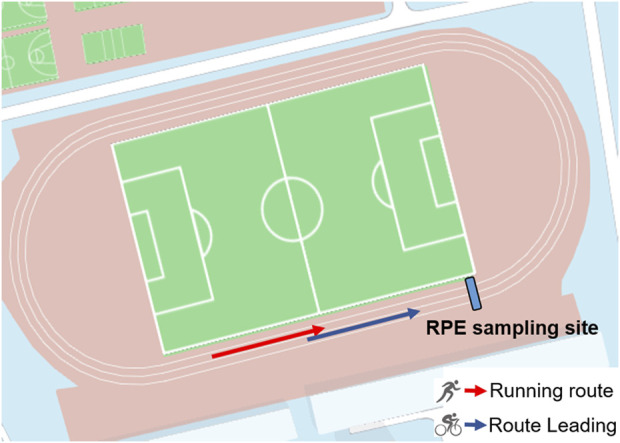
Top view of the test area.

The test protocol was implemented on a standard athletic track (Figure 1) and fully explicated to the subjects before fatigue test. The use of Borg RPE 6–20 scale (Borg, 1970; Scherr et al., 2013) was explained to the subjects prior to the experiment. Prior to formal protocol all subjects performed a warm-up of 2 laps of jogging, followed by any necessary stretching was performed. The running fatigue test consisted of three consecutive parts.

(Ⅰ) The first test was performed according to the fatigue protocol, which involved running 10 laps at a constant speed for a total distance of 4,000 m in the first lane. An electric bicycle was used in the second lane to control the speed, using the average speed of the individual’s best 10 km performance in the previous 6 months. Meanwhile, the rider was required to keep the speed as constant as possible. The RPE scale was recorded every 400 m through verbal questions and answers.
(Ⅱ) In the second test, a core process of fatigue was implemented. The runners entered it from the first test without interval, the initial speed of the second test was the same as the first test, and then increased by 0.2 km/h every 100 m. The protocol was applied by using electric bicycle guidance as first test. The speed was increased at the four intersections of straight and curved lanes. The RPE value was recorded every 100 m. The speed increment was continued until the participant was unable to follow or the RPE value was greater than 16 (very difficult) (Marotta et al., 2021). Due to inter-individual differences in physical ability, participants completed different distances in this part.
(Ⅲ) The third test was transited from the second test consecutively, in which the speed was reduced so that to keep as same as the first test. The total running distance in the third part was 1,200 m. RPE value was recorded every 400 m.

**FIGURE 2:**
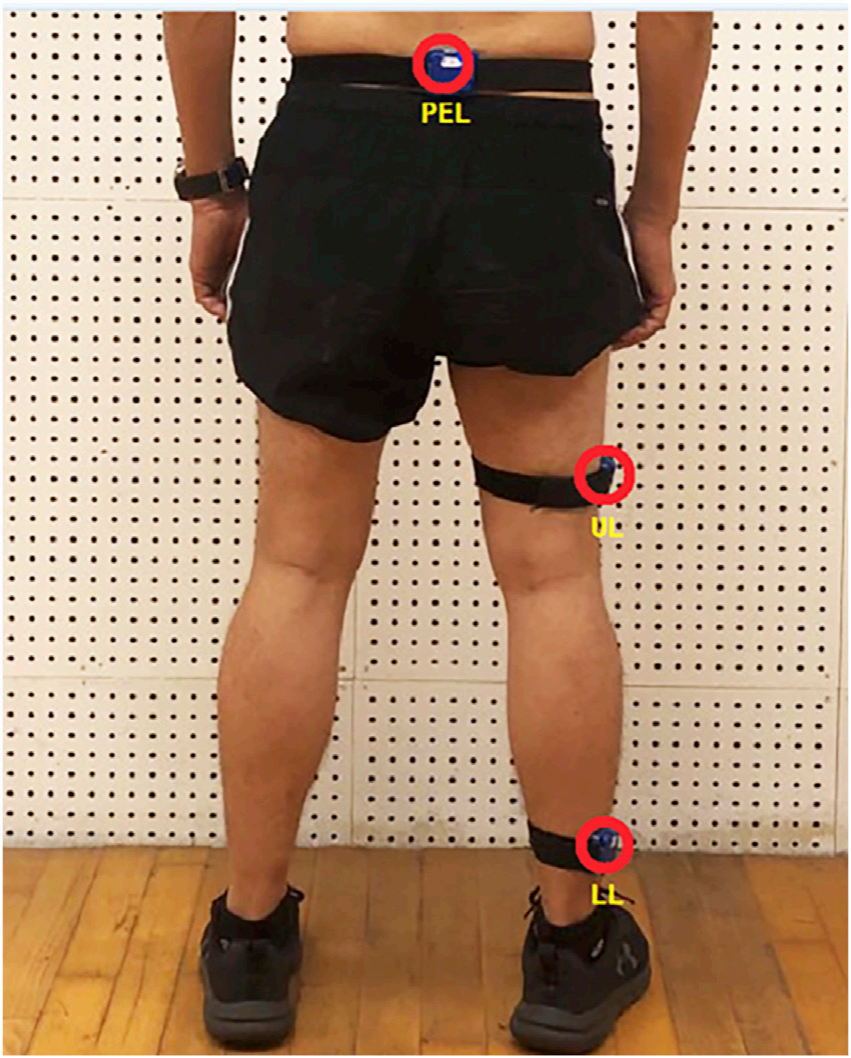
Measurement setup of inertial sensors (LL for lower limb, UL for upper part of lower limb and PEL for pelvis).

During the test, a Garmin sports watch (Fenix Chronos, Garmin, United States) was used to record and display the running pace, speed and heart rate (HR) information. One physiotherapist attached the IMUs to pre-determined specific anthropometric locations (Figure 2) on the participant. Nylon elastic bands with pocket buckles were used to fix the three IMUs to the subject’s right lower limb (LL), upper part of lower limb (UL), and pelvis (PEL) positions. The shank IMU was placed 2 cm above the lateral ankle condyle (marginal distance), the thigh IMU was placed at the lateral part of the mid-lower thigh, and the pelvis IMU was placed at the fifth lumbar vertebra. The orientation and location of the IMUs was consistent across all subjects.

### 2.2 Fatigue stage identification

**FIGURE 3:**
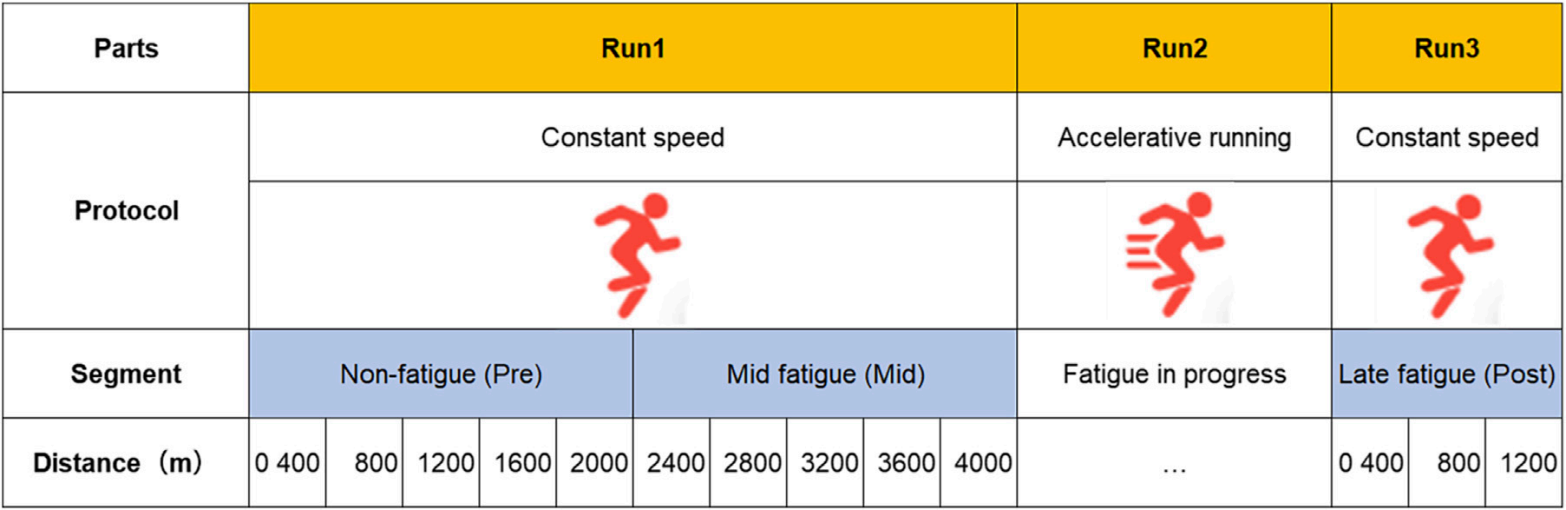
Test program process and fatigue stage division.

In order to deeply understand the generation and development of fatigue, according to the division method of Marotta et al. (2021), the first part was divided into two stages: the first 2,000 m was defined as the initial fatigue stage (Pre), the second 2,000 m was defined as the middle fatigue stage (Mid), and the 1,200 m of the third part after the acceleration running intervention was defined as the late fatigue stage (Post). In the experimental design, the running speed was the same in the three stages. In the construction of the dataset, the interval was divided according to RPE, with the early fatigue data intercepted in the first stage, the mid-fatigue data intercepted in the second stage, and the post-fatigue data intercepted in the 1,200 m after the acceleration. Based on the principle that individual RPE values did not overlap between the three stages, the detailed division of each stage is shown in Figure 3. This deep learning model took the raw IMU data as input, without dividing the gait cycle, and directly segmented the labeled intercepted data according to a constant-length window.

### 2.3 Data processing and statistics

Three WT901SDCL IMUs (Wit-motion Technologies, Shenzhen, China) were used for data acquisition. All IMU ranges were set as: triaxial accelerometer (±16 g), gyroscope (±500°/s), and magnetometer (±12 Ga). Based on the Shannon sampling theorem and the Nyquist criterion, a sampling frequency of 200 Hz was chosen. To avoid time-base errors between different IMUs, the IMUs were calibrated before each test and frame synchronization was performed with the local acceleration generated by the vertical straight knee drop landing (Mitchell et al., 2015). A Kalman filter was used to estimate the 3D orientation by fusing the accelerometer, gyroscope, and magnetometer data (George et al., 2020). The raw accelerometer and gyroscope data were low-pass filtered using a fourth-order Butterworth filter with a cut-off frequency of 15 Hz (O'Reilly et al., 2017).

Data were expressed as mean ± standard deviation (M±SD), and one-way analysis of variance (ANOVA) was performed using SPSS 26.0 statistical software (SPSS Science, Chicago, United States) for data in the early, middle and late stages of fatigue, and the significance level was set at *α* = 0.05.

### 2.4 Raw time series selection

The deep model uses the raw time series data as input for validation, and the data directly sampled from the IMU include the tri-axis acceleration (ACC) (ax, ay,az) and the tri-axis angular velocity (GYR) (gx,gy,gz). To facilitate the model calculation, the Kalman filtering method is used to calculate the tri-axis positional angle (POS) (px,py,pz), i.e., the complete time series at any time (t) can be expressed as follows:

$$x=\left[\text{ACC}\left(t\right),\text{GYR}\left(t\right),\text{POS}\left(t\right)\right]$$ (1)

The dataset includes data from all subjects in the fatigue test. The input of each time series tries to contain signals of more than one complete gait cycle (Tran et al., 2021). That is, the intercept criterion for constructing the input series is as follows:

$$N_{c}\geq f_{s}T$$ (2)

Where $N_{c}$ is the intercept length, $f_{s}$ is the IMU sampling frequency, and T is the gait cycle, whereby the time length is set to 200 consecutive sampling points (i.e., 1 s of data), which is sufficient to cover a complete gait cycle.

To achieve ideal prediction results, deep neural networks generally require a large amount of data as support (Poulose et al., 2022). When dividing the original data, different overlap degrees are set for data segmentation. Specifically, two methods are used to construct the data set, both of which use sliding windows for data interception with a window length of 200 and overlap settings as follows:

First, the segmentation was performed using no overlap (Overlap was set to 0%, the step length was 200) to obtain a 0% Overlap dataset without overlap, with a total number of 17,174 data subsets.

Then, half of the window length was used as the overlap (Overlap was set to 50%, the step length was 100) for segmentation, and the 50% Overlap dataset with 50% overlap was obtained, and the number of subsets in the dataset was 34,334.

In this study, a random shuffle operation (shuffle) was performed on the dataset. Using a 9:1 ratio, 90% of the full data set was used as the training set and the remaining 10% was used as the test set. The training set was then divided again using an 8:2 ratio, 72% of the full data set was used as the training subset and 18% as the validation set.

To investigate the effects of accelerometer and gyroscope on model prediction, three different data combinations were used to compare the effects of different training inputs on fatigue prediction results in the same dataset, based on acceleration and gradually adding angular velocity and Eulerian angles, as described in Table 1.

**TABLE 1:** Time series dataset information description.

Dataset name	Data selection (dimension)	Subset length	Split overlap (%)	Number of subsets
0%Overlap	ax, ay, az	200	0	17,174
	ax, ay, az, gx, gy, gz	200	0	17,174
	ax, ay, az, gx, gy, gz, px, py, pz	200	0	17,174
50%Overlap	ax, ay, az	200	50	34,334
	ax, ay, az, gx, gy, gz	200	50	34,334
	ax, ay, az, gx, gy, gz, px, py, pz	200	50	34,334

Note: ax, ay, az refer to the tri-axis acceleration, gx, gy, gz refer to the tri-axis angular velocity, and px, py, pz refer to the tri-axis attitude angle.

**FIGURE 4:**
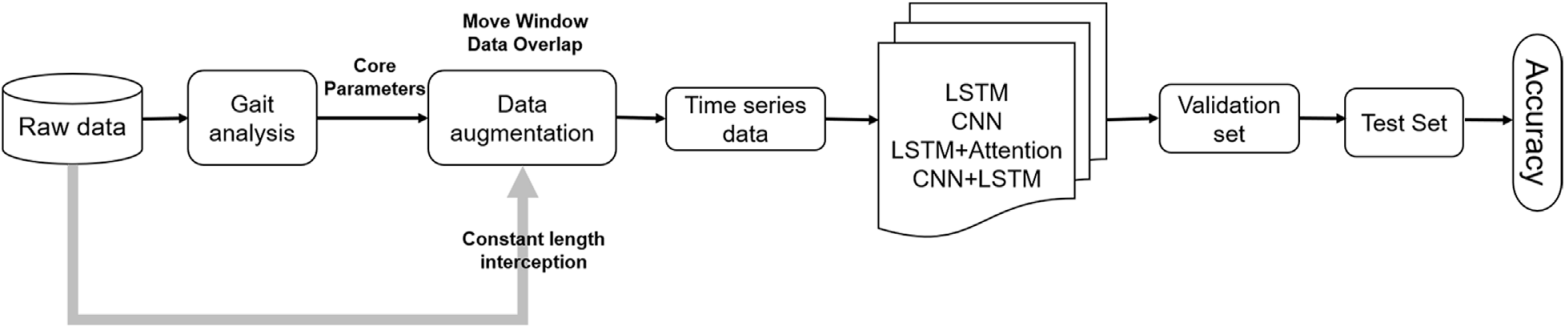
Flow of data processing.

The overall flow of data computation is shown in Figure 4, where the deep learning model is completed using the raw time series of the constant-length dataset.

### 2.5 Deep learning model

In the study of fatigue classification using raw time series as input, the effects of different deep learning neural network models were compared. Specifically, single-layer LSTM (S_LSTM), CNN, dual-layer LSTM (D_LSTM), single-layer LSTM plus attention model (LSTM + Attention), CNN and LSTM hybrid model (LSTM + CNN) are used.

The LSTM adapts two different models for the raw data sequence, which utilizes a sequential structure that linearly stacks multiple network layers (Schmidhuber, 2015). Comparing the differences of different neural network architectures of one or two LSTM layers, in order to unify the scattered data, the data is batch standardized after the LSTM layer, and then a dropout layer is added to prevent overtraining (overfitting), and finally a fully connected dense layer is added to limit the weights and avoid too large. The weights in this layer are regularized, and the output of this layer is also regularized. The unit number of LSTM parallel hidden layers is set to 128, and the input dimension input_shape is set to (200, input_dim), where 200 represents the length of the input sample sequence, which is actually one second of data according to the sampling frequency. Input_dim is the dimension selected for the data. To avoid overfitting during model training, the neural network units are temporarily removed from the network during training according to a certain probability ratio, that is, a Dropout layer is added after the LSTM layer with a Dropout ratio of 0.5. The fully connected dense layer is added after the Dropout layer, and softmax is used for the activation function, L2 regularization is used, and the constraint ratio is 0.01. The maximum number of training iteration epochs is set to 200 and the batch size is set to 256. Meanwhile, the model loss function loss is tracked by the EarlyStopping mechanism, and training is stopped early after 14 iterations without loss reduction.

Specifically, the CNN model consists of four convolutional layers and two pooling layers (Ma et al., 2022). The convolutional layer extracts data features along the time series direction, while the pooling layer is used to calculate local sensitivity and perform secondary feature extraction and calculation. The pooling layer maintains the feature invariance while reducing the feature dimension (reducing the number of parameters to be optimized), and using global max pooling instead of average pooling. Finally, the output results of the convolutional layer and the pooling layer are converted to dimensions, and the multi-dimensional input is one-dimensional (Flatten layer) and input into the fully connected layer (Dense layer). The activation function used by the convolutional layer in CNN is ReLU, while the activation function used by the fully connected layer is softmax. Table 2 shows the specific structure parameters of the CNN.

**TABLE 2:** Structure of CNN model with ACC + GYR as input.

Layer (type)	Size	Output shape	Param #
conv2d (Conv2D)	18*1	(None, 100, 6, 32)	608
max_pooling2d (MaxPooling2D)	2*1	(None, 50, 6, 32)	0
conv2d_1 (Conv2D)	9*1	(None, 50, 6, 64)	18,496
conv2d_2 (Conv2D)	3*1	(None, 50, 6, 128)	24,704
max_pooling2d_1 (MaxPooling2D)	2*1	(None, 25, 6, 128)	0
conv2d_3 (Conv2D)	1*6	(None, 25, 1, 128)	98,432
flatten (Flatten)	--	(None, 3,200)	0
dense (Dense)	--	(None, 3)	9,603

Total params: 151,843; Trainable params: 151,843; Non-trainable params: 0.

The dual-layer model structure is similar to the single-layer LSTM structure, the first LSTM layer with the number of hidden layers unit is still set to 128, the input dimension is the same as the single-layer structure, the difference is that the first LSTM layer can return a 3D tensor as input for the later layers, a second LSTM layer is added after the first layer (the number of hidden layers unit is set to 64), and a dropout layer is added after each layer. The fully connected Dense layer configuration is the same as the single-layer model. The same regularization, learning rate scheduling, and early termination mechanisms are used in training.

In the single-layer LSTM plus attention model, the calculation based on the attention mechanism mainly includes the following two parts: first, the attention distribution of all the input information is calculated, and then the weighted average calculation of the input information is completed according to the distribution (Lin et al., 2021). Set the input as:

$$X=\left[{x_{1,}\cdots ,x}_{n}\right]$$ (3)

Given a task related query vector ***q***, computed under given ***q*** and **x**, select a particular input vector of probability is expressed as:

$$\alpha_{n}=softmax\left(s\left(x_{n},q\right)\right)=\frac{\exp\left(s\left(x_{n},q\right)\right)}{\sum_{j=1}^{N}\exp\left(s\left(x_{j},q\right)\right)}$$ (4)

Where *α*_n is the attention distribution, a softmax-like computational method is introduced to numerically transform the scores so that the data can be normalized and the originally computed scores can be transformed into a probability distribution with the sum of all element weights equal to 1. s (**x, q**) is the attention scoring function used to compute the similarity or correlation between the two. For the specific calculation, additive, dot product, and bi-linear calculation methods can be used. This model is computed using the dot product with scaling correction as follows:

$$s\left(x,q\right)=\frac{x^{T}q}{\sqrt{D}}$$ (5)

Where D is the dimensionality of the input vector and plays the role of scaling correction. When the dimensionality *D* of the input vector is relatively high, the result of the dot product usually has a larger variance, which leads to a smaller gradient of the softmax function, and the scaling-corrected dot product model can solve this defect.

Finally, by weighted averaging, the attention distribution *α* can be interpreted as the degree of attention (weight) of the α_n input vectors x_n for a given task-related query, and the input information is aggregated using the information selection mechanism to obtain the attention value:

$$\text{Attention}\left(x,q\right)=\sum_{n=1}^{N}\alpha_{n}x_{n}$$ (6)

Layer LSTM combined with attention model is to add attention mechanism based on single-layer LSTM, that is, add attention layer after dropout layer of LSTM, and the detailed structure is shown in Table 3.

**TABLE 3:** Structure of LSTM plus Attention model.

Layer (type)	Output shape	Param #
lstm (LSTM)	(None, 200, 128)	70,656
dropout (Dropout)	(None, 200, 128)	0
last_hidden_state (Lambda)	(None, 128)	0
attention_score_vec (Dense)	(None, 200, 128)	16,384
attention_score (Dot)	(None, 200)	0
attention_weight (Activation)	(None, 200)	0
context_vector (Dot)	(None, 128)	0
attention_output (Concatenat)	(None, 256)	0
attention_vector (Dense)	(None, 128)	32,768
dense (Dense)	(None, 3)	387

Total params: 120,195; Trainable params: 120,195; Non-trainable params: 0.

Consider that the data of the three axes of the accelerometer are expressed with certain internal connections (constraints) at the same moment, and that the data of the accelerometer and gyroscope also have a certain correlation. Therefore, when the data are presented, the six axes of IMU data constitute the temporal data in the vertical time axis and also the series in the horizontal direction. This allows either CNN or LSTM to process the data structure, taking into account the temporal and spatial information of the signal. When constructing the model, the original signal array is passed through LSTM and CNN respectively to learn the temporal and spatial features, and the feature expression ability is enhanced by information fusion.

**FIGURE 5:**
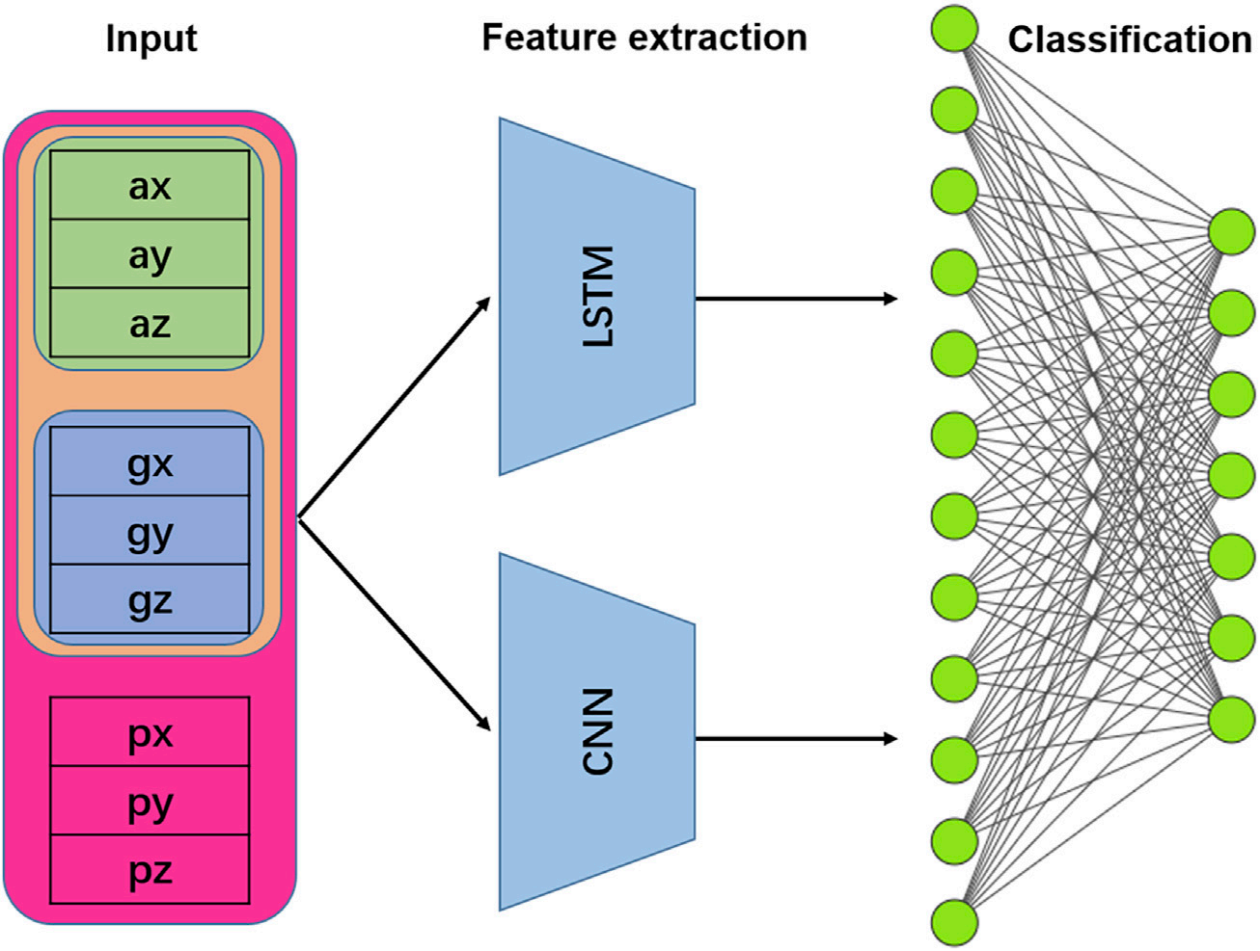
Structure of the LSTM + CNN hybrid model. Note: ax, ay, az refer to the tri-axis acceleration, gx, gy, gz refer to the tri-axis angular velocity, and px, py, pz refer to the tri-axis attitude angle.

In this study, the LTSM in the CNN and LSTM hybrid model adopts the single-layer LSTM network introduced above, and the CNN adopts the same structure as the independent CNN model to learn features separately. The features learned by the two models are concatenated in parallel, and then input to the following layer (fully connected layer) for recognition. The structure is shown in Figure 5.

The output feature vectors of LSTM and CNN are concatenation, and the combined results are input into the fully connected layer. Finally, the softmax function is used to complete the model prediction, and the fully connected layer in all models is described as follows:

$$f\left(x\right)=\sum wx+b$$ (7)

$$\hat{y}=\text{softmax}\left(f\left(x\right)\right)$$ (8)

Where $f\left(x\right)$ is the output of the previous layer, w is the weight, b is the bias, and $\hat{y}$ is the output of softmax. Based on this, the cross-entropy loss function is constructed:

$$loss=-\frac{1}{n}\sum p\left(x\right)lnq\left(x\right)$$ (9)

Where n is the number of classifications, p is the true value, and q is the predicted value.

All models were trained using TensorFlow2.0 deep learning framework, Python version 3.7.9, Pandas and Numpy packages were used for basic data operation and processing, and the deep model was built using Keras learning package. The computer configuration uses Intel dual-core i7-6600U (2.60GHZ), 8 GB memory, graphics card NVIDIA GeForce 930M.

## 3 Results

### 3.1 Comparison of fatigue stages

The fatigue RPE values and HR statistics for the three stages of the experiment are shown in Table 4.

**TABLE 4:** Three stage RPE values and HR (times/min).

Stages	Pre	Mid	Post
RPE	6.6 ± 0.8	11.9 ± 1.1	15.2 ± 1.5
HR	163.2 ± 4.9	171.8 ± 5.0	178.0 ± 4.8

The subjects were not counted because of inconsistencies in distance and duration of running during the acceleration to fatigue. The RPE values for the three stages presented an upward trend, and a one-way ANOVA indicated that the comparison of data between any two categories was statistically significant (*p* < 0.05). For the HR data, the comparison of data between the three stages had a significant difference (*p* < 0.05).

### 3.2 Performance of the deep learning models

A single IMU was used to construct the dataset based on the raw data series with different constructions. The results of the fatigue classification accuracy in different models are shown in Table 5.

**TABLE 5:** Classification accuracy of different models based on raw IMU data (Accuracy, %).

Name of data set	Data source	S_LSTM	CNN	D_LSTM	LSTM + Attention	LSTM + CNN
LL 0%Overlap	ACC	85.39	86.50	88.94	88.53	88.16
	GYR	84.92	92.78	87.19	85.10	93.19
	ACC + GYR	89.35	89.41	90.57	90.57	89.64
	ACC + GYR + POS	96.80	97.32	96.68	97.15	**97.67**
LL 50%Overlap	ACC	92.46	95.69	93.94	95.22	96.59
	GYR	89.43	95.86	92.87	92.37	95.69
	ACC + GYR	93.45	96.21	94.82	95.31	95.66
	ACC + GYR + POS	98.17	99.33	99.13	99.18	**99.62**
UL 50%Overlap	ACC	89.55	89.08	91.50	92.46	89.90
	GYR	86.86	92.69	88.09	90.65	92.72
	ACC + GYR	90.19	90.91	93.16	93.30	90.77
	ACC + GYR + POS	95.95	97.57	97.18	**98.51**	98.34
PEL 50%Overlap	ACC	87.16	86.60	88.53	90.01	89.43
	GYR	85.73	91.88	89.46	88.61	91.47
	ACC + GYR	90.36	90.36	89.81	92.46	91.44
	ACC + GYR + POS	96.85	96.97	96.74	**97.29**	97.09

Note: LL, low limb sensor; UL, upper part of lower limb sensor; PEL, pelvis sensor; ACC, tri-axis acceleration; GYR, tri-axis angular velocity; POS, tri-axis pose angle.

Using a single LL IMU data, the training results of different models on the two datasets showed that the 50% overlap dataset outperformed the 0% overlap dataset overall for fatigue classification using the same model approach, with a 4.51% difference in the lowest accuracy value and a 1.95% difference in the highest accuracy value between the two datasets.

Comparing the classification effects of different models, the dual-layer LSTM model was better than the single-layer LSTM model, while the LSTM + Attention model was better than the dual-layer LSTM model; the multi-layer CNN model had a slightly higher recognition effect than the dual-layer LSTM model; the hybrid LSTM + CNN model and LSTM + Attention had similar accuracy, and the highest classification accuracy appeared in these two models. In terms of data interception, compared to using ACC in three directions as input, increasing the input dimensions improved the prediction accuracy of most models. In the CNN model, the superposition of UL and LL IMU, ACC and GYR did not bring any improvement. The magnitude of improvement brought by adding POS was higher than that brought by adding GYR. The minimum accuracy of all models was 84.92% and the maximum accuracy was 99.62%.

Comparing the classification results using ACC alone with those using GYR alone, the classification effect of ACC was higher than that of GYR in the LSTM model, whether it was single-layer LSTM, dual-layer LSTM, or LSTM + Attention. In the CNN model, the classification effect of ACC was inferior to that of GYR, and in the LSTM + CNN model, the classification effect of ACC was close to or inferior to that of GYR. For example, the classification accuracy of IMU in LL was 3%, 1%, and 3% higher than that of GYR in the single-layer LSTM, dual-layer LSTM, and LSTM + Attention models, respectively, while in the CNN and LSTM + CNN models, the classification accuracy of GYR was slightly higher than that of ACC.

Comparing the classification accuracy of ACC + GYR and GYR, the performance of different models varied greatly by adding GYR based on ACC. And the single-layer LSTM showed the greatest improvement in performance, with almost 5% improvement over PEL. However, in the CNN model, the data of PEL and UL showed that the former was even 1.52% lower than the latter, and the recognition accuracy of mixed data on CNN + LSTM did not improve.

The recognition accuracy of different parts of IMU was compared, and based on individual IMU, the recognition rate of LL was the highest, which was significantly better than that of UL and PEL. Using the raw data of ACC and GYR as input, the recognition rate of UL was 96.21%, which was higher than 92.46% for PEL IMU (Table 5).

**FIGURE 6:**
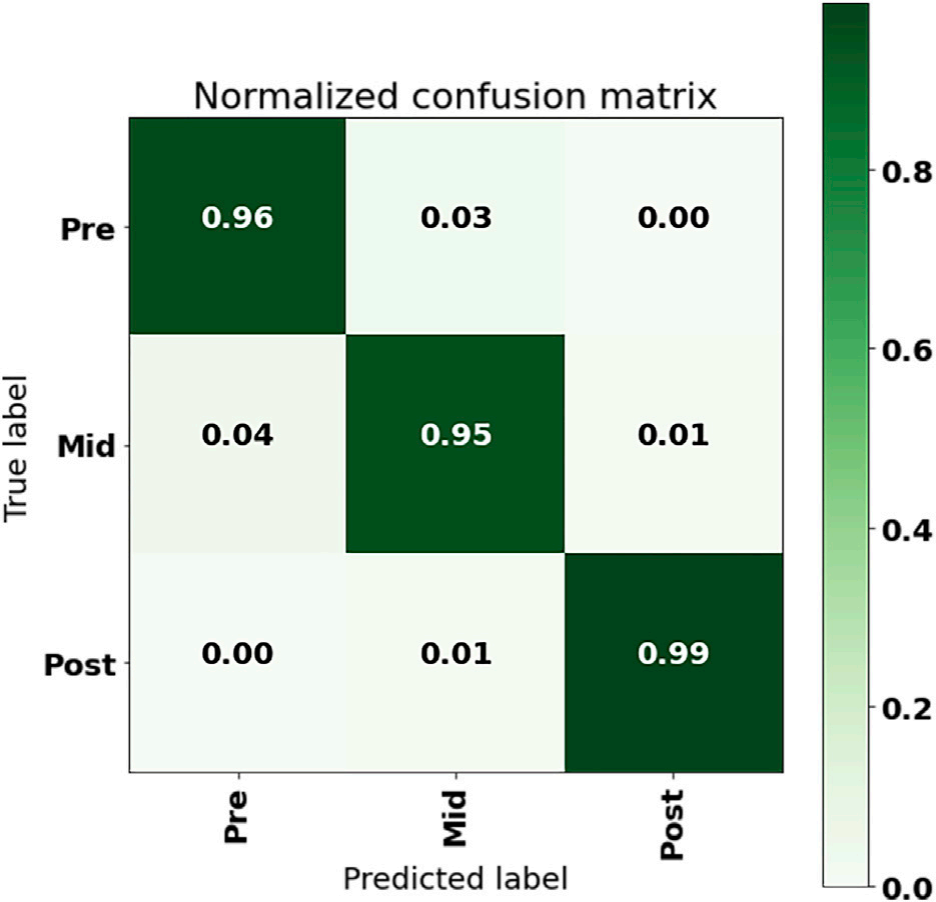
Confusion matrix of LL IMU with ACC + GYR input to CNN.

The confusion matrix of the CNN model with ACC + GYR as input from the 50% overlap data set using a single sensor of the LL is shown in Figure 6. From the results, it could be seen that the model prediction error points out the confusion of pre-fatigue and mid-fatigue data, that is, about 3% of the pre-fatigue period data was falsely predicted as the mid-fatigue period data, while about 4% of the mid-fatigue data was falsely predicted as the pre-fatigue data. The incorrect intersection of mid-fatigue and late-fatigue data was about 1% of the total data.

**FIGURE 7:**
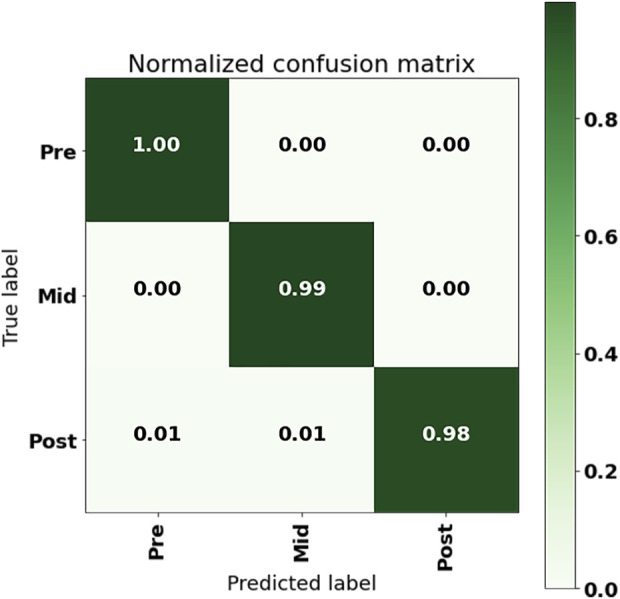
Confusion matrix of LL IMU with ACC + GYR + POS input to LSTM + Attention.

The confusion matrix of the LSTM + Attention model output using the 50% overlap data set with a single sensor in the LL, with ACC + GYR + POS as input, is shown in Figure 7. From the confusion matrix, it could be seen that the model prediction error occurred when the real post-fatigue was predicted as pre-fatigue and mid-fatigue, that is, about 1% of the post-fatigue data was falsely predicted as pre-fatigue, and about 1% of the post-fatigue data was falsely predicted as mid-fatigue.

## 4 Discussion

The RPE statistics of the three stages showed that the RPE data gradually increased with the increase of exercise duration and the difference between the three stages was obvious. Although the running speed of the subjects remained constant during the three periods of data collection, the heart rate after accelerated running was significantly higher in the third stage than in the second phase (*p* < 0.05). Combined with the RPE values, the efficacy of the accelerated running intervention in producing exercise fatigue was further confirmed.

The use of wearable devices to identify and predict human fatigue is a recent research focus. Previous studies have shown that fatigue impairs cognition and affects motor performance, which reduces movement efficiency and increases the risk of injury, thus objective measures of fatigue are critical in areas such as occupational health and safety (Adão Martins et al., 2021). The ability of wearable devices to continuously monitor biomedical signals for a long-term period in an unattended environment, while being non-invasive and comfortable, provides us with a very powerful solution for fatigue monitoring. In our study, by comparing the fatigue classification effect of the two datasets with different overlaps, it can be seen that the performance at 50%Overlap is higher than that at 0%Overlap using the same model, with a maximum difference in accuracy of 9.19% and a minimum difference in accuracy of 1.95% for the two datasets. It indicates that setting half of the moving window length as the step overlap (50% Overlap) effectively improves the performance of the model while expanding the capacity of the dataset, which is consistent with the findings of some previous studies using IMU that data enhancement of the IMU dataset allows the model to characterize the data information more deeply, improving the generalization ability of the model and avoiding overfitting (Tran et al., 2021; Mitchell et al., 2015).

Zou et al. (2020) showed that the contribution of the acceleration signal in the IMU contributed more to the recognition than the angular velocity signal from the gyroscope, and it concluded that the accelerometer was superior to the gyroscope for characterizing gait characteristics. In this study, the fatigue classification results of the PEL IMU data showed that using the same model with only gyroscope data and only acceleration data as input, the former had an overall significantly better classification effect than the latter. It can be speculated that in gait individual recognition, the weight of segment acceleration may be higher than the segment swing, so the acceleration recognition effect is better than gyroscope. While in studying the effect of fatigue on human body, the change of the amount of information related to link swing bearing may be greater than the change of information of impact buffer, resulting in the gyroscope recognition effect is better than accelerometer.

In terms of data inputs for fatigue prediction, the inclusion of the GYR signal improved the model performance, indicating that the effect of fatigue on running is not only reflected in impact, but also in limb oscillation and twisting. The inclusion of the POS signal in the input substantially improved the discrimination of the fatigue level, indicating that the IMU posture corresponding to the limb changes the action pattern in running. In the selection of data sets, the study on the recognition of abnormal gait patterns of the lower limbs showed that different signal composition of the IMU would have different effects on the model. For example, Kiprijanovska et al. (2020) used the IMU in a smartwatch with data from accelerometers, gyroscopes and magnetometers as inputs in independent or different combinations, and showed that the combination of accelerometers and gyroscopes can improve the accuracy of a single accelerometer from 85.8% to 87.8%, and the model accuracy is also significantly higher with three sensors fused as input than the results with only two sensors.

Differences in exercise protocols may contribute to differences in performance between data sets. Different exercise regimens produce fatigue by different mechanisms, which may also affect the biomechanical performance of fatigue. O'Reilly et al. (2017) performed fatigue tests using the Leger standard test method, in which subjects performed progressive strides (with increasing cadence) on a 20 m track according to a signal cadence. And Karvekar et al. (2021) used a fixed frequency squatting protocol to induce fatigue.

This study adopted a fatigue exercise protocol similar to that of Marotta et al. (2021) was used, with a before-and-after comparison of constant speed running followed by the addition of variable speed running with incremental loading and then a return to the original constant speed running. Obviously, there are some differences in muscle recruitment as well as energy utilization between progressive folding running and squatting protocols, with the former being closer to a whole-body process of transition from aerobic to anaerobic exercise, whereas the latter focuses on fatigue of localized muscles in the thighs. Although, according to the definition of exercise fatigue, it is determined when the physiological processes of the body cannot continue to be performed at a certain level and/or cannot maintain the booked exercise intensity during the exercise, due to the difference in energy metabolism between constant speed running and variable speed running, the fatigue of the former is often related to the inhibition of energy reserve utilization processes, while the latter is clearly characterized by intra-muscle cell metabolic changes leading to a decrease in the rate of ATP conversion (Ament and Verkerke, 2009). Therefore, these differences may also lead to different patterns of changes in lower limb movement, resulting in differences in the classification effect of fatigue.

Currently, many deep learning studies use CNN models that focus more on extracting spatial information, while the temporal characteristics of the gait cycle are equally important from a gait perspective (Tran et al., 2021). This study proposes a relevant LSTM-based model for fatigue recognition, which can bypass the conventional idea based on gait cycle segmentation and, instead, use a direct constant-length (time) interception method. Moreover, the accurate segmentation of gait cycles is inherently a challenging task that is influenced by the IMU wearing position, wearing firmness and gait event detection algorithms. The accuracy of gait cycle segmentation will undoubtedly have an impact on subsequent detection, as it is often accompanied by large noise during interception and constant-length transformation. The results of this study showed that the LSTM and CNN based models can extract the hidden features of gait sequences without synchronizing the sequence signals, and achieve the classification of different fatigue levels.

The number of layers of the LSTM determines the number of training model parameters, and the more layers, the more parameters (Xue et al., 2022). A single-layer model tends to imply a simple model structure, but is not flexible enough and easily underfitted, while the probability of overfitting increases as the number of model layers increases, and too many layers can easily weaken the generalization ability of the model. The comparison of the fatigue prediction results from single-layer LSTM and dual-layer LSTM showed that the increasing the number of layers improves the prediction accuracy. This study demonstrated that the dual-layer LSTM can perform the recognition task well by using raw IMU data as input. In this study, in addition to the single-layer LSTM model and the dual-layer LSTM model, a hybrid model based on LSTM and Attention is proposed, which can further reduce the confusion in the recognition of adjacent motion stages and thus improve the fatigue prediction performance. The attention model has two advantages: first, it can reduce the computational burden of processing high-dimensional data sets by structurally selecting a subset of the input, which can effectively reduce the dimensionality of the data. Second, it allows the model to focus more on finding the correlation between the input data and the current output, thus improving the performance of the output. The ultimate goal of the attention model is to help the network better learn the interrelationships between multiple content modalities so that it can better represent this information and overcome its inability to explain the drawbacks of a more difficult design (Lim and Zohren, 2021). From the results of this study on fatigue prediction based on IMU time series data, it can be seen that the attention mechanism is suitable for the inter-map relationships between different modal data, which may be difficult to interpret, hidden and complex in IMU data, but it does not affect the expression of the model. In short, the introduction of the attention mechanism can make the LSTM model more effective for the processing of IMU data.

Temporal information of human motion can provide important information for action patterns recognition, and CNN focuses more on data features at specific time points, which may reasonably ignore some information (Ji et al., 2013). The LSTM used in this paper is a typical recurrent neural network (RNN) model, and the two mechanisms work independently and then merge in the hidden layer, which can better extract the features of the temporal signal. The good performance of CNN and LSTM hybrid models can be interpreted as CNNs extract spatial features from local time regions, while LSTMs focus on the overall (long-term) features, and CNN models alone have better discrimination than LSTMs, and their classification performance is even close to that of hybrid models. Davidson et al. showed that CNN models perform best for classification of time-series data (Davidson et al., 2020), which is also similar to the findings of Jiang et al. (2021b) that the deep ConvLSTM model performs similarly to CNNs. Compared with RNN models, which are commonly used for classification of time-series data, the convolutional layer of the CNN is better able to learn the deep features contained in recursive patterns. Since the input of the deep model is raw IMU data, which is sequential data that contains both temporal and spatial information, the importance of CNN for fatigue prediction can be affirmed accordingly. In addition, the introduction of Attention or CNN in the model, while improving the performance of the model for fatigue recognition, has the advantage of being executed in a more time-efficient manner compared to the multi-layer LSTM model. Because an important shortcoming of multi-layer LSTM is that the model training process takes too long to compute (Balaji et al., 2021), while the introduction of Attention and CNN can effectively reduce the number of training parameters, thus making the network training process of more efficient.

Using the raw IMU data as input, the IMU fatigue classification results from different parts of the IMU showed that the LL IMU also performed much better than the PEL IMU. This is consistent with the results of Marotta et al. (2021), whose study found only 61% recognition accuracy for the lumbar region, but 76% for the calf. However, it is not consistent with the results of O'Reilly et al. (2017), whose study found 75% identification accuracy for the lumbar region, but only 67%–70% accuracy for the calf. The prediction models used in both studies were random forest models, and both used feature engineering as input for fatigue prediction and classification. Furthermore, Karvekar et al. (2021) also used feature engineering to classify gaits with different stages of fatigue through support vector machine algorithm and reported that the classification accuracy was only 78%. In contrast, in this study, raw data was used for segmentation and directly as input for training. In terms of classification results, the accuracy of all deep learning models based on raw data can reach more than 85%, which may obtain better classification results than feature engineering.

This study also found that the classification results for the LL IMU were significantly better than the UL IMU, while the UL IMU outperformed the PEL IMU. Consistent results were obtained regardless of whether a dual-layer LSTM, CNN, or LSTM + CNN model was used. The difference in classification results with ACC as input reached 5%–9%, while the difference was reduced to 2%–3% with the three signals as input. From the perspective of biomechanics, due to the role of human skeletal muscle system, the upward transmission process of ground impact is an attenuation process, so the closer to the ground, the more information it contains about the impact, reflecting the swinging and braking information of the distal extremity of the lower limbs. Certainly, because the IMU is attached to a unilateral limb, it contains less information about the symmetry of the lower limb. In contrast, the lumbar IMU reflects relatively more symmetry information, but it contains relatively less motor control and impact information of the terminal limb.

From the classification differences of IMU in different parts, it can be seen that the unilateral limb has a higher degree of discrimination than the lumbar site, that is, in the process of running to fatigue, the biomechanical influence of fatigue on the periodic movement of the lower limb terminal segment is higher than its influence on the center of gravity. Considered from the perspective of motor control, it can be explained that the human body regulates and controls the foot-ground interaction pattern to maintain the relative stability of the upper limb (including the trunk) by absorbing the impact of the distal limb after fatigue.

## 5 Limitation

There are also some limitations in this study. First, in this experiment, the IMU is fixed by nylon elastic band, and the relative movement with the human body is small. The direction of the IMU relative to the fixed segment is consistent for all subjects during the test, but it is difficult to require this in practical applications, so the subsequent research needs to consider the direction of IMU, and increase the noise interference caused by the IMU rotation, and improve the generalization ability of the neural network model. Second, although the samples were intercepted with constant-length, since the samples did not cover multiple continuous gait cycles, this study only considered the variation within the gait cycle, and still did not consider the absolute long-range relative relationships during the gait cycle, such as gait stability, symmetry, and variability. Future research can start from these perspectives and even consider the long-range nonlinear characteristics of gait to construct better predictive classification models. Additionally, although this study used a single sensor with the advantages of easy wearability and low cost, from a biomechanical point of view, this may lose a large amount of information, while more useful information is bound to be beneficial to improve the accuracy of fatigue classification. Future research may consider multi-sensor fusion evaluation to enhance its practical application value in exercise fatigue supervision.

## 6 Conclusion

The IMU-based wearable sensor enables the possibility of continuous measurement of exercise fatigue in a realistic environment, using a single IMU that can accurately distinguish between different levels of running fatigue states. Based on this dataset, this study proposes a deep learning model with constant-length interception of the raw IMU data as input. Both CNN and LSTM can effectively complete the classification of fatigue IMU data, the attention mechanism can effectively improve the processing efficiency of LSTM on the raw IMU data, and the hybrid model of CNN and LSTM is superior to the independent model, which can better extract the features of raw IMU data for fatigue classification. This study will provide some reference for many future studies of movement patterns based on deep learning.

## Data Availability

The original contributions presented in the study are included in the article/Supplementary Material, further inquiries can be directed to the corresponding authors.
